# In-Depth Analysis of the Structure and Properties of Two Varieties of Natural Luffa Sponge Fibers

**DOI:** 10.3390/ma10050479

**Published:** 2017-04-29

**Authors:** Yuxia Chen, Na Su, Kaiting Zhang, Shiliu Zhu, Lei Zhao, Fei Fang, Linyan Ren, Yong Guo

**Affiliations:** College of Forest and Garden, Anhui Agricultural University, Hefei 230036, China; sheherose@163.com (Y.C.); suna122216@163.com (N.S.); 15905690096@163.com (K.Z.); zhuslwood@163.com (S.Z.); 15077921608@163.com (L.Z.); ffei_1988@163.com (F.F.); 15755139436@163.com (L.R.)

**Keywords:** luffa sponge fiber bundles, mechanical properties, anatomical characteristics, moisture regain, thermal performance

## Abstract

The advancement in science and technology has led to luffa sponge (LS) being widely used as a natural material in industrial application because of its polyporous structure and light texture. To enhance the utility of LS fibers as the reinforcement of lightweight composite materials, the current study investigates their water absorption, mechanical properties, anatomical characteristics and thermal performance. Hence, moisture regain and tensile properties of LS fiber bundles were measured in accordance with American Society for Testing and Materials (ASTM) standards while their structural characteristics were investigated via microscopic observation. Scanning electron microscopy (SEM) was used to observe the surface morphology and fractured surface of fiber bundles. The test results show that the special structure where the phloem tissues degenerate to cavities had a significant influence on the mechanical properties of LS fiber bundles. Additionally, the transverse sectional area occupied by fibers in a fiber bundle (S_F_), wall thickness, ratio of wall to lumen of fiber cell, and crystallinity of cellulose had substantial impact on the mechanical properties of LS fiber bundles. Furthermore, the density of fiber bundles of LS ranged within 385.46–468.70 kg/m^3^, significantly less than that of jute (1360.40 kg/m^3^) and *Arenga engleri* (950.20 kg/m^3^). However, LS fiber bundles demonstrated superior specific modulus than *Arenga engleri*.

## 1. Introduction

**Figure 1:**
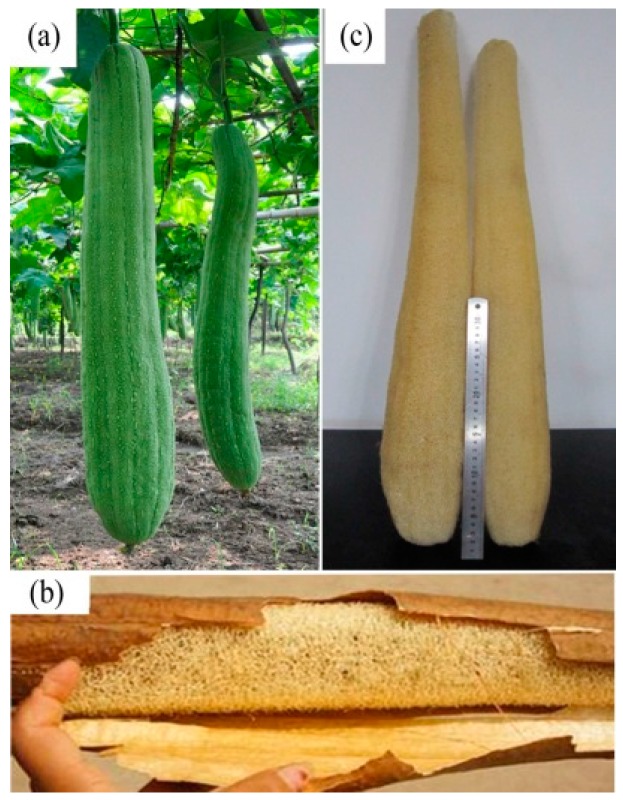
(**a**) Luffa cylindrical; (**b**) luffa sponge with cortex; and (**c**) luffa sponge.

**Figure 2:**
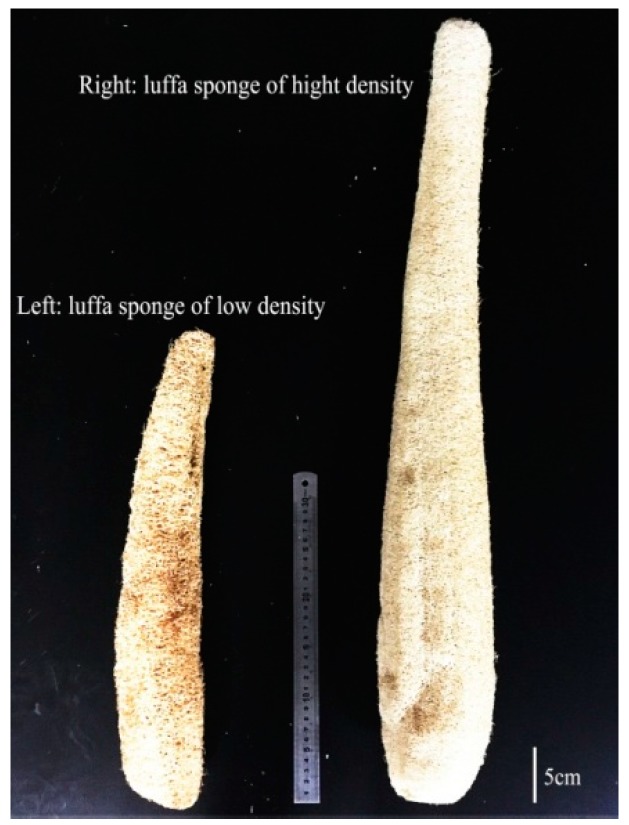
The luffa sponge of low density (**left**); and high density (**right**).

The luffa sponge (LS), commonly referred to as vegetable sponge or luffa cloth, is obtained from matured dried fruit of luffa cylindrica. It consists of crisscrossed fibers and presents a three-dimensional reticular structure. Photographs of luffa cylindrica and luffa sponge are shown in Figure 1. The luffa sponge is a rich resource for plants and is cultivated in the tropical countries of Asia and Africa, and some sub-tropical areas. The plantation areas in China include Jiangxi, Henan, Sichuan, Guangdong, Jiangsu, Anhui, and a few other places [1,2,3]. The luffa sponge is a low-density, eco-friendly resource that is non-toxic, biodegradable, and has stable physical and chemical properties [4,5]. There are two common varieties of luffa sponge, namely, luffa sponge of low density and luffa sponge of high density (as shown in Figure 2), among which the luffa sponge of high density is the improved variety [6]. The luffa sponges have a lengthy history of being studied in China. The Chinese classic “Compendium of Materia Medica” recorded about the luffa performance 400 years ago and specified that “luffa sponge, with sweet and clod nature, has the effect of clearing heat, cooling blood, detoxifying, promoting circulation of blood and dredging collaterals”. The luffa sponge has been used as cleaning equipment (for instance, as a dish cloth), insole materials, and pillow filling materials. The advancement in science and technology has led to luffa sponges being widely used in several fields such as pharmaceutical engineering [7,8], environmental engineering [9,10,11], biotechnology [12,13,14], and industrial products [15,16,17]. Since luffa sponges are porous material with a high degree of lignification [18,19,20], they have great potential for applications in composite materials and fabric fibers.

Of particular interest when studying the luffa sponge fibers and their applications in composite materials are the water absorption and mechanical properties of sponge gourd–polyester composites, luffa sponge cellulose, and nanometer composite material, and the structure and mechanical strength of luffa sponge fiber bundles. Boynard et al. investigated water absorption and mechanical properties of sponge gourd–polyester composites [21]. The results demonstrated that, in comparison with general plant fiber reinforced composites, luffa sponge reinforced composite material has greater moisture absorption and lesser mechanical strength. However, these properties can be improved if a barrier layer avoids a close contact between the fiber and the external environment. Hence, it is expected that water absorption in luffa sponge fiber would have a great impact on the behavior of sponge gourd–polyester composites. However, the water absorption properties of luffa sponge fibers have not received significant attention until now. In 2010, Siqueira et al. investigated the behavior of luffa sponge nanocomposites and compared the mechanical properties of luffa cylindrical micro fibrillated cellulose (MFC) and whiskers films [15]. The results showed that the degree of crystallinity has a significant impact on mechanical properties of luffa sponge nanocomposites. In addition, in 2012, Shen et al. studied the structural strength of luffa sponge column and indicated that the structural strength has a close relationship with density of luffa sponge column [4]. Chen et al. performed a multi-scale study to understand the relationship between the structural and mechanical properties at different levels of its hierarchical organization (the fiber of inner layer, foam-like blocks of lateral position and different forms columns) and different orientations, including longitudinal direction, tangential direction, and radial direction. The results showed that the inner layer fibers of hoop wall make significant contributions to longitudinal properties of luffa sponge column followed by the tangential properties, while the core part has lesser mechanical strength than inner layer [22]. One possible explanation of the results could be the variable porosity distribution in the luffa sponge, having a lower porosity in the hoop wall and higher porosity in the core part. However, the mechanical strength of different parts and the effect of density on mechanical strength have not been investigated yet.

Moreover, a significant number of studies have shown that, as the reinforcing phase of polymer composites, mechanical strength of fibers has a significant impact on the mechanical properties of polymer composites [23,24]. Zhai et al. have reported that the structural characteristics of diameter of fiber bundles, transverse sectional area occupied by fibers in fiber bundles, transverse sectional area occupied by vessels and phloem tissue in fiber bundles, diameter and wall thickness of fiber cell, microfibril angle, and the degree of crystallinity have a significant influence on the mechanical properties of windmill palm fiber bundles. In summary, it is essential to conduct an in-depth study about moisture absorption, mechanical properties, and structural characteristics for enhanced understanding of luffa sponge reinforced composite materials. In the current work, the moisture absorption, mechanical properties, structural characteristics, and the thermal performance of fiber bundles in four layers of luffa sponge for both high density (31 kg/m^3^–65 kg/m^3^) and low density (15 kg/m^3^–30 kg/m^3^) LS have been studied.

## 2. Materials and Methods

### 2.1. Sample Preparation

**Figure 3:**
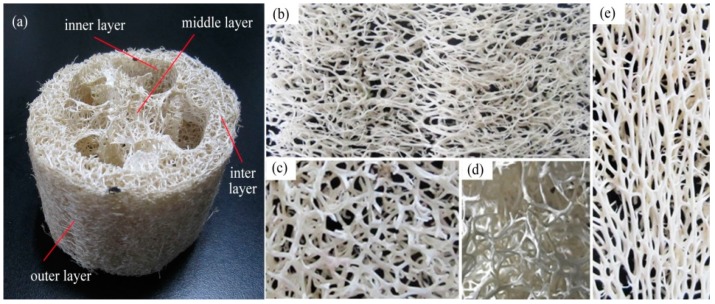
The geometrical features of luffa sponge column: (**a**) different regions; (**b**) orientation of outer layer; (**c**) orientation of inter layer; (**d**) orientation of middle layer; and (**e**) orientation of inner layer.

**Figure 4:**
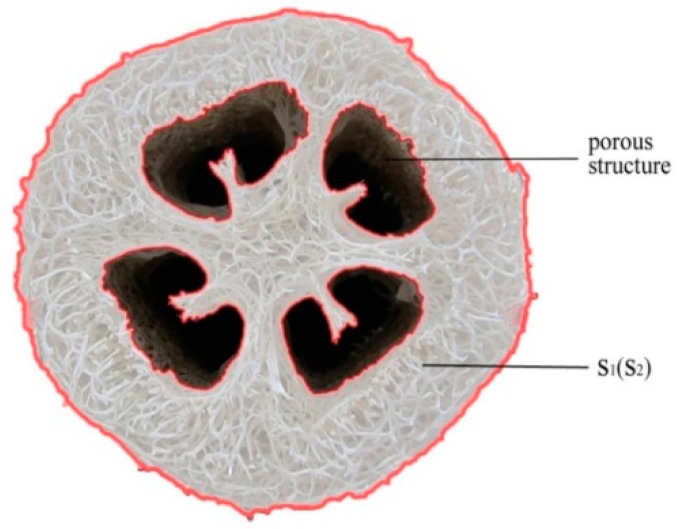
The transverse section of luffa sponge column.

To account for aging contraction, the two ends of luffa sponge were amputated and the middle sections of luffa sponge columns (Figure 3a), which are similar to cylindrical ones, were selected. Before collection, luffa sponge columns were rinsed several times in rinsing water and dried. Subsequently, the luffa sponge columns were placed in humidity chamber at 21 °C and 65% relative humidity (RH) for 24 h. The humidity chamber was set up using distilled water and held at room temperature. The density of luffa sponge columns was calculated using Equations (1) and (2):

(1) $$\rho =\frac{m}{v}$$

(2) $$v=\frac{\left(s_{1}+s_{2}\right)\times 2}{H}$$

where *ρ* is density of luffa sponge columns in kg/m^3^, M is the mass of luffa sponge column in kg, and *v* is the volume of luffa sponge columns in m^3^. S_1_ and S_2_ are the areas of the top and bottom transverse section from luffa sponge column (excluding the porous structure on the transverse section), respectively. The transverse section of luffa sponge column is shown in Figure 4. The area is expressed in m^2^, while H, the height of luffa sponge column, is expressed in m.

Luffa sponge, on the basis of density, can be classified into two categories; namely, LD and HD. The density range of 15 to 30 kg/m^2^ is considered as low density, while the density range of 31 to 65 kg/ m^2^ is considered as high density.

As shown in Figure 3, each luffa sponge column is composed of an outer, inter, middle, and inner layer. The eight groups of single fiber bundles (length ~30 mm) were obtained from four layers in the luffa sponges of two densities for tensile strength test, moisture regain (MR) determination, and surface morphology observation. To gather significant data, the luffa sponge column and luffa sponge fiber bundles were randomly selected. The single fiber bundles of the same length were obtained from jute and *Arenga engleri* for contrast test. In addition, the eight groups of short fiber bundles (length ~2 mm) were obtained from luffa sponge to prepare frozen microtome sections.

The fiber bundles of four layers from luffa sponge of two densities, jute, and *Arenga engleri* were dried in an air oven at 100 °C for 24 h, and, thereafter, eight kinds of powder specimens were obtained by ball mailing for X-ray diffraction test (XRD) and thermogravimetric analysis test (TGA). The speed of ball mailing used was 170 turns/min while the ball mailing duration was kept at 5 min.

### 2.2. Microscopic Observation and Imaging Quantification

Subsequent to section collection, diameters of fiber bundles from luffa sponge, jute, and *Arenga engleri* were measured via digital optical microscope (Nikon Corporation, Nikon Eclipse E100, Tochigi , Japan).

To observe the morphological characteristics of fiber cells in fiber bundles and measure the length of fiber cells in luffa sponge, five to ten fiber bundles were bound tightly together by cotton threads and cut into lengths of 20 mm. Subsequently, the bundles were segregated in solution of 1:1 glacial acetic acid and hydrogen peroxide for duration of 8 h. Thereafter, fiber cells were rinsed several times until neutral pH was reached. A digital optical microscope was used to observe .morphological characteristics of fiber cells. The length measurements were carried out using MIAS image analysis software. To gather significant statistical data, 90 fiber cells from each group were tested.

To observe the micromorphological characteristics of transverse sections of fiber bundles, eight groups of fiber bundles (length ~2 mm) from luffa sponge were embedded in tissue freezing medium. The temperature of frozen embedding ranged from 23 to 13 °C below zero. The tissue freezing medium was offered by Amos Scientific Pty. LTD. (Melbourne, Australia). Thereafter, frozen microtome sections of 10 µm thickness were cut from embedded specimens using a freezing microtome (Hestion Co., Ltd., CM3800, Shanghai, China). A few of the transverse sections were stained with safranin in order to clearly observe the lignified tissue via digital optical microscope. The diameters of fiber cell, and the thickness and diameter of fiber cell wall were measured via Motic Images, an image analysis software.

**Figure 5:**
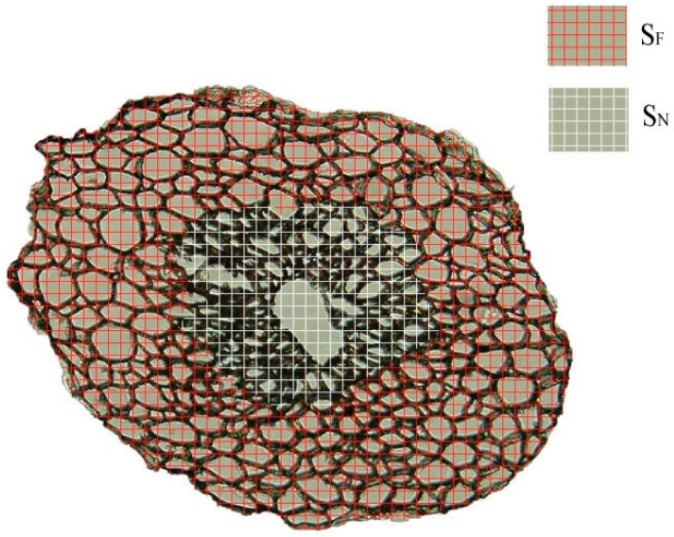
Areas of S_F_ (transverse sectional area occupied by fibers in a fiber bundle) and S_N_ (transverse sectional area occupied by non-fibers )in a fiber bundle of Luffa sponge.

The other sections were observed under a polarized-light microscope (Olympus Corporation, BX51, Tokyo , Japan) to obtain the area of transverse section (S), the amount of transverse sectional area occupied by fiber in a fiber bundles (S_F_), and the amount of transverse sectional area occupied by non-fibers including cavity, vessels, and phloem tissue (S_N_). The S_F_ and S_N_ are shown in Figure 5. To obtain statistically significant number of samples, 20 sections for each layer of luffa sponge were observed. The microscopes equipped with LCD Olympus video camera (model DP70, Olympus Corporation, Tokyo, Japan) were used to resolve images and Motic Images image analysis software was used to conduct measurements.

### 2.3. Tensile Measurements

**Figure 6:**
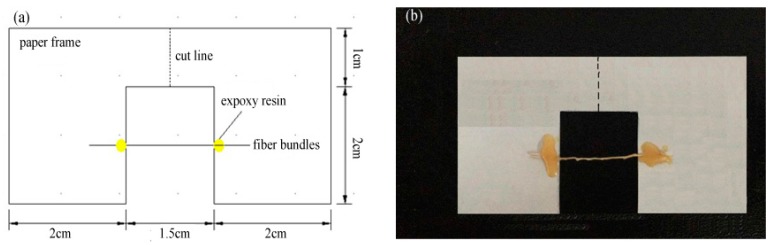
(**a**) The paper frame support used for tensile strength tests; and (**b**) a single fiber bundles is fixed on the paper frame by means of epoxy adhesive. The paper is then cut in two along the dotted line and the paper supports are pulled apart.

For the tensile strength measurements, the fiber bundles of luffa sponge, jute, and *Arenga engleri* were air-dried till their moisture content ranged from 8% to 10% by weight (wt %) and thereafter cut into sections of 30 mm in length. All the tensile tests were conducted on the universal testing machine (Shimadzu Corporation, Shimadzu AG-X Plus, Kyoto, Japan) with a 1 KN load cell and the crosshead speed of 0.5 mm/min. The testing room conditions were around 25 ± 2 °C with a RH of 70%. To immobilize fiber bundles, in accordance with ASTM D 3379-75 standard [25,26,27,28], a 15 mm gauge length paper frame using two part epoxy adhesives was used in our study (Figure 6a). Prior to tensile measurements, the middle part of the supporting paper frames was cut as shown in Figure 6b. To obtain effective statistical data, 50 fibers from each group were tested. Furthermore, the mean diameter of each fiber bundle was measured using digital optical microscope (magnification = 200×) at ten random points along the gauge length. As discussed by Munawar et al. [25,27], mean diameter was used to calculate fiber’s transverse sectional area, which is a vital parameter for determining fiber tensile strength. Finally, the fractured fiber bundles were collected and fractured surfaces were observed via scanning electron microscope (Hitachi Co., Ltd., S-4800, Tokyo, Japan).

### 2.4. Moisture Regain

In accordance with ASTM D 2654-89a standard, bundles of single fibers bound together were weighed and dried in an air oven at 105 °C for 4 h. The samples were weighed using a balance with accuracy to four decimal places (±mg) and recorded as *M_d_*. The samples were thereafter placed in humidity chamber at 65% RH at 21 °C for 24 h [29,30]. To obtain statistically significant number of samples, three repetitions were carried out for each test and twenty fibers samples were randomly selected for each test. Subsequently, the fibers were weighed again after exposure to humidity and recorded as *M_h_*. Finally, *MR* was determined using Equation (3):

(3) $$MR=\frac{M_{h}-M_{d}}{M_{d}}\times 100\%$$

where *M_h_* is the mass of the sample after humidity exposure, and *M_d_* is the mass of the dried sample.

### 2.5. X-ray Diffraction Analysis

The crystallinity index of the tested fibers was calculated from X-ray Diffraction patterns recorded on the XRD-3 X-ray diffractometer (Beijing Purkinje General Instrument Co., Ltd., XRD-3, Beijing, China). The diffractograms were obtained by varying angle (2θ) from 10° to 40°. To obtain statistically significant number of samples, three repetitions were carried out for each datum and mean of the data was selected. The crystallinity index (*C_r_*I) of the fiber was calculated using Equation (4) [31,32]:

(4) $$C_{r}I=\frac{I_{002}-I_{am}}{I_{002}}\times 100\%$$

where *C_r_*I is relative crystallinity index, *I*_002_ is the maximum intensity of diffraction of the (002) lattice peak at 2θ angle between 22° and 23°, and *I_am_* is the intensity of diffraction of the amorphous material which is taken at 2θ angle between 18° and 19° when the intensity is minimal.

### 2.6. Thermo-Gravimetric Analysis (TGA)

To evaluate thermal stability of luffa sponge fibers, TGA was carried using TG209 thermogravimetric analyzer (Netzsch Corporation, TG209, Goliaths, Germany). Samples with weight between 5 and 10 mg were placed in an alumina pan and heated from 30 to 700 °C. To prevent oxidation, TGA analysis was performed under nitrogen atmosphere at a flow rate of 20 mL/min. The heating rate was maintained at 10 °C/min during heating between 30 and 700 °C, apart from keeping at the temperature at 100 °C for 10 min [33]. To obtain statistically significant number of samples, three repetitions of each test were carried out.

## 3 Results and Discussion

### 3.1. Structure of Fiber Bundles

**Figure 7:**
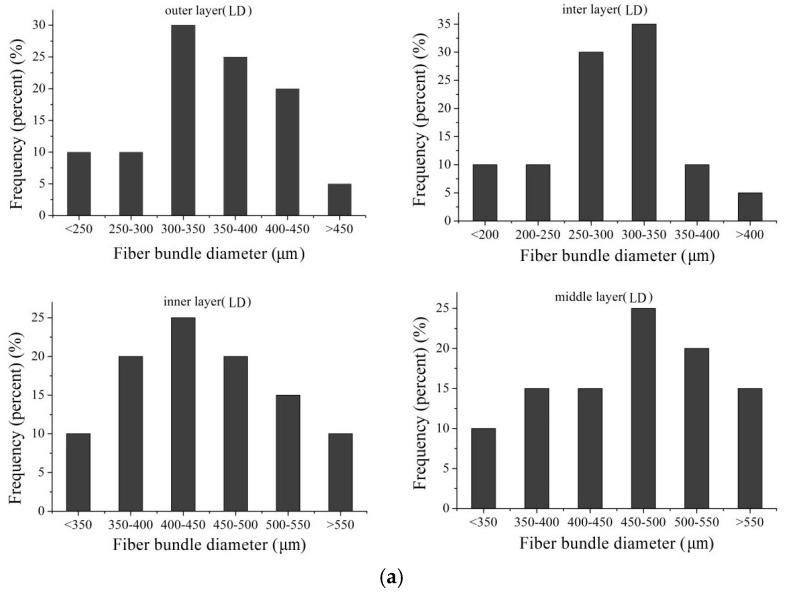

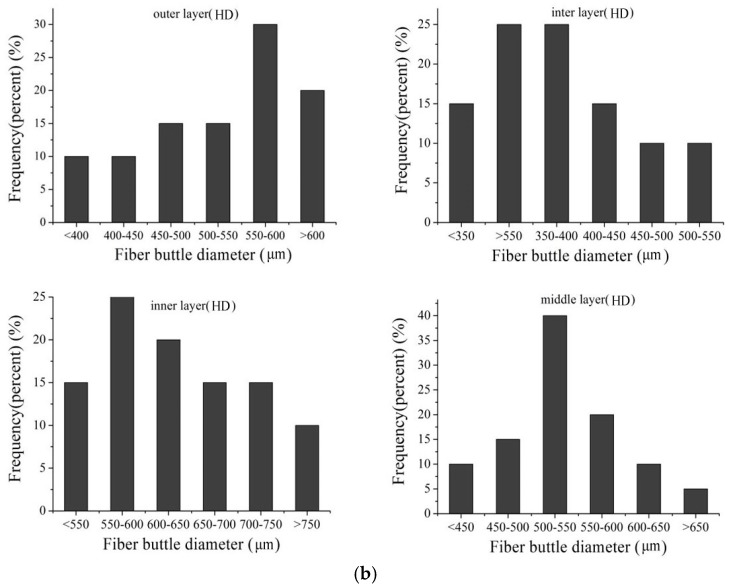
The diameter distributions of fiber bundles: (**a**) taken from luffa sponge of low density; and (**b**) taken from luffa sponge of high density.

**Figure 8:**
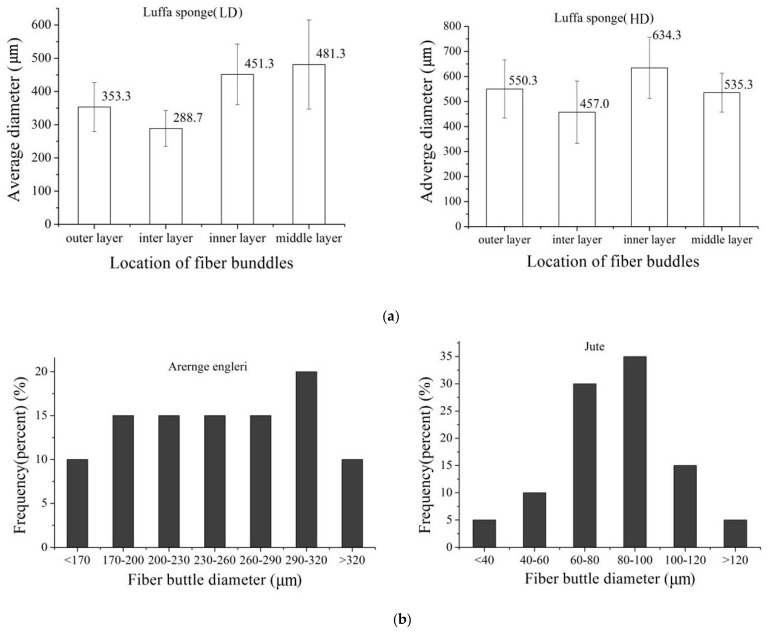
(**a**) The average diameters of fiber bundles taken from luffa sponge; and (**b**) diameter distribution of fiber bundles taken from *Arernge engleri* and jute.

The difference in the diameters of fiber bundles of low density luffa sponge, high density luffa sponge, jute, and *Arenga engleri* was investigated (Figure 7 and Figure 8). The diameters of four layers from the same density range were different. In the case of LD luffa sponge, the fiber bundles in middle layer and inner layer have larger diameter followed by the outer layer and the inter layer. Specifically, for LD luffa sponge, the diameters of fiber bundles in the outer, inter, inner, and middle layer were 353.3 µm, 288.7 µm, 451.3 µm, and 481.3 µm, respectively. However, in the case of HD luffa sponge, the diameter of fiber bundles was the largest in the inner layer, followed by the outer layer and the middle layer, and the smallest in the inter layer. Specifically, for HD luffa sponge, the diameters of outer, inter, inner and middle layer were 550.3 µm, 457.0 µm, 634.3 µm, and 535.3 µm, respectively. It is to be noted that that there is a slight difference in diameters of middle layer fiber bundles between LD and HD luffa sponges; on the contrary, there is a significant difference in diameters of fiber bundles in the hoop wall (including outer layer, inter layer, and inner layer). The mean diameter of fiber bundles in the hoop wall of LD luffa sponge was about 200 µm larger than that of HD luffa sponge. The structural division of the sponge is shown in Figure 3. Each layer of the sponge has its unique feature. The outer layer is composed of thin fibers with the orientation roughly surrounding the sponge (Figure 3b). The fiber bundles of inner layer running along the vertical axis are straight (Figure 3e). The inter layer consists of a complex branched fiber network (Figure 3c). In comparison with the hoop wall, fibers of the core part are loosely interconnected with each other, and a thick single fiber runs along the central line of the complete luffa sponge (Figure 3d) [4].

In addition, the diameters of fiber bundles from *Arenga engleri* were distributed in the range of 170 to 320 µm while those of jute had a relatively more concentrated distribution in the range of 40 to 120 µm. Zhai et al. reported that mean diameters of outer layer from sheath of windmill palm is 345 µm, while those of middle layer and inner layer are 418 µm and 202 µm, respectively. The diameters of Sisal aggregates (*Agava sisalana*) have been reported to be in the range of 100–400 µm while the typical diameter of coir fiber bundles from the coconut palm (*Cocos nucifera*) has been reported to be around 200 µm [34,35]. Hence, the fiber bundles of luffa sponge have larger diameters than common plant fibers.

**Figure 9:**
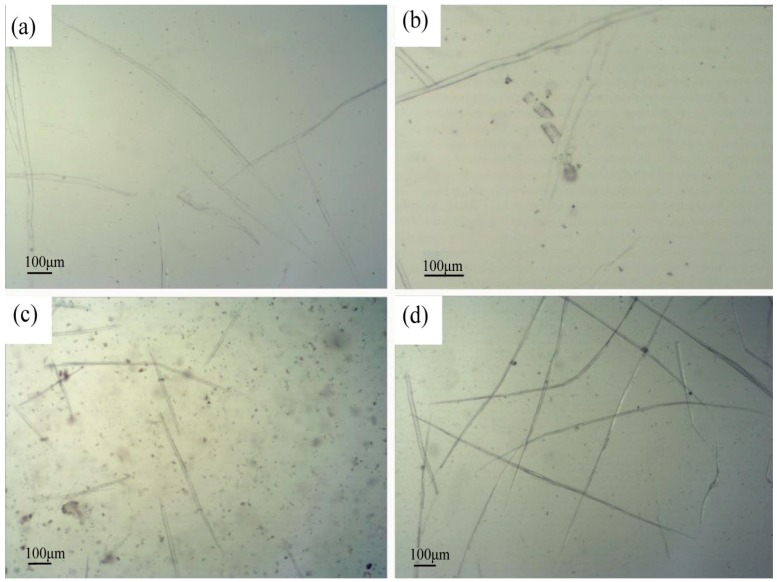
The morphology of fiber cells: (**a**) fiber cells of luffa sponge; (**b**) threaded pipe of luffa sponge; (**c**) fiber cells of *Arenga engleri*; and (**d**) fiber cells of Jute.

The morphology and characteristics of fiber cells from luffa sponge, *Arenga engleri* and jute are presented in Figure 9 and Table 1. The fiber cell of luffa sponge had a shuttle-like shape and was easy to bend. It had a characteristic length in the range of 1050–1070 µm, diameter in the range of 17–28 µm, lumen diameter in the range of 12–24 µm, and wall thickness in the range of 3–8 µm. There was a slight difference in length and diameter between LD and HD luffa sponges. Nevertheless, the wall thickness and ratio of wall to lumen of fiber cell from HD luffa sponge were larger than that for LD luffa sponge (excluding the inter layer).

Secondly, the fiber cell of luffa sponge was longer than that of *Arenga engleri*, but shorter than that of the jute. The ratios of wall to lumen for luffa sponge were significantly smaller than that of *Arenga engleri* and jute. Thus, it can be concluded that in comparison with other common plant fiber cells, the fiber cell of luffa sponge contains less material substance, which is the primary reason for lightness of luffa sponge. Furthermore, the ratio of wall to lumen of fiber cells in inner layer of LD and HD luffa sponges was 0.20 and 0.39, respectively, and is slightly larger than that of other layers.

**Figure 10:**
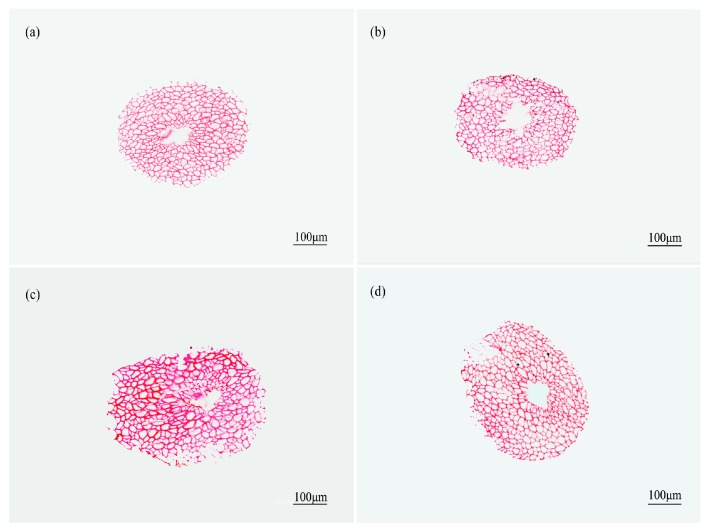
Transverse sections of fiber bundles obtained from luffa sponge of low density from: (**a**) outer layer; (**b**) inter layer; (**c**) inner layer; and (**d**) middle layer.

**Figure 11:**
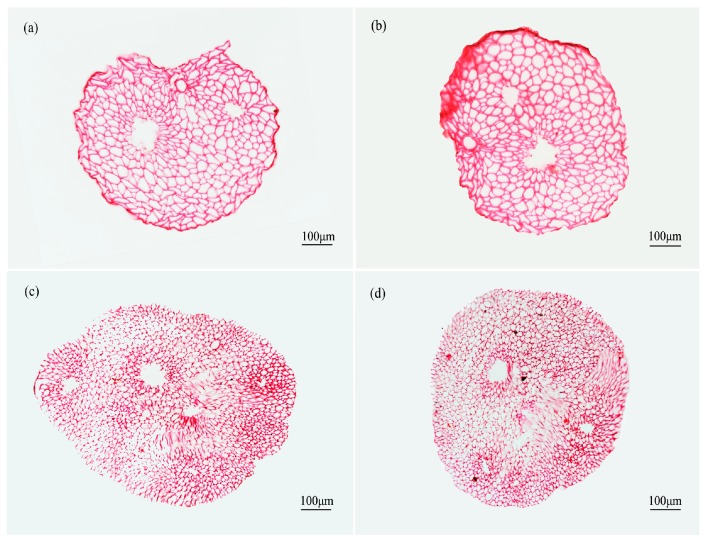
The transverse sections of fiber bundles obtained from luffa sponge of high density from: (**a**) outer layer; (**b**) inter layer; (**c**) inner layer; and (**d**) middle layer.

**Figure 12:**
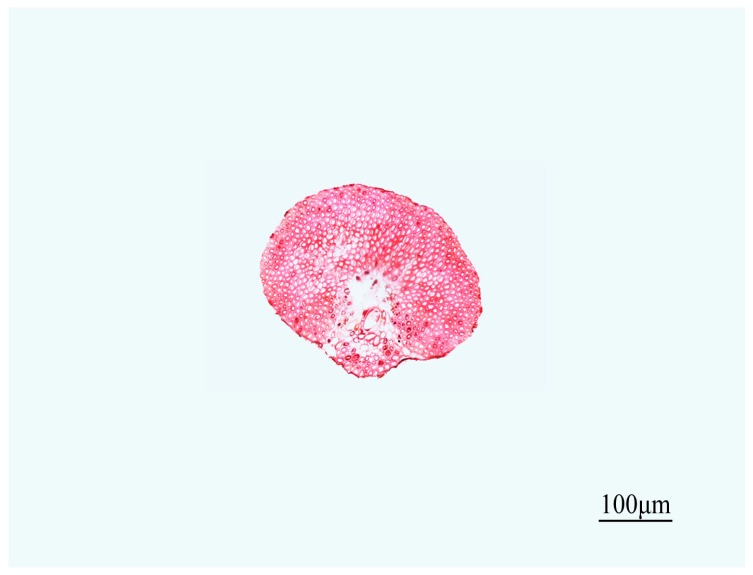
The transverse section of fiber bundles taken from *Arenga engleri*.

The transverse sections of the safranin-stained fiber bundles clearly revealed lignified tissue. According to Figure 10, Figure 11 and Figure 12, the diameters of fiber bundles from HD luffa sponge were significantly larger than that of LD luffa sponge. Besides, the fiber bundles from *Arenga engleri* had smaller diameters than those of luffa sponge. It can be observed that fiber bundles of luffa sponge primarily consisted of fiber cells. As shown in Figure 10 and Figure 11, there were cavities, with a small number of vessels and phloem tissue nearby, around the center of fiber bundles [36]. However, as shown in Figure 12, similar phenomenon could not be observed in the fiber bundles of *Arenga engleri*. Moreover, it was also discovered that there were two or more cavities with uneven distribution in the fiber bundles of HD luffa sponge, while there was only one cavity around the center of fiber bundles in LD luffa sponge.

**Figure 13:**
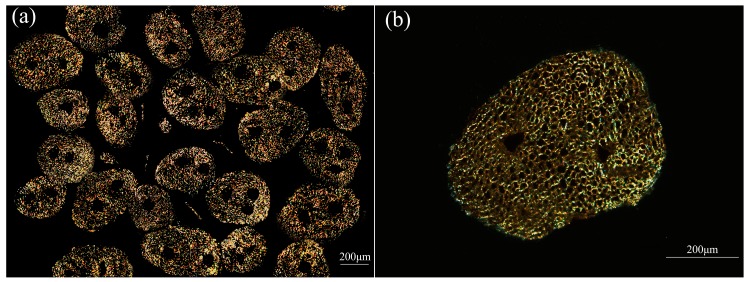
Transverse section image of fiber bundles taken fromHD luffa sponge and observed by polarized-light. (**a**) is the transverse section image in low-magnification times, and (**b**) is in a high magnification times. (the noticeable dark region is the area occupied by non-fiber including cavity, vessels, and phloem tissue).

Figure 13 shows transverse sectional images of the fiber bundles of luffa sponge as observed in the dark field by polarized-light microscope. We could clearly observe the dark regions in the transverse section of each individual fiber bundle. In addition, we observed that two or more dark regions existed in the fiber bundles of HD luffa sponge, while only one dark region existed near the center of the fiber bundles from LD luffa sponge. The dark regions were proved to be the cavities, vessels, and phloem tissue.

Hence, S, S_F_, and S_N_ of a fiber bundle were measured for each of the four layers of luffa sponge. The statistical data are shown in Table 2, where S_F_% is the ratio of S_F_ to S, while S_N_% is the ratio of S_V_ to S. The S_N_% of luffa sponge is in the range of 13–28%. The S_N_% of fiber bundles of corresponding layer in HD luffa sponge is larger than that of LD The value of S_N_% represents the immaterial substance in the fiber bundles; in other words, the lower is the value of S_N_%, the higher is the amount of substantial fibers in fiber bundles. Zhai et al. have reported that the amount of substantial fibers has an important influence on the mechanical properties of fiber bundles [25].

In addition, variance analysis results of characteristic index values of fiber bundles of luffa sponge are shown in Table 3. The data indicated that S and S_N_% of fiber bundles from four layers in low density (or high density) luffa sponge were significantly different. Meanwhile, there was a significant difference in S and S_N_% of fiber bundles between LD and HD.

### 3.2. Mechanical Properties

**Figure 14:**
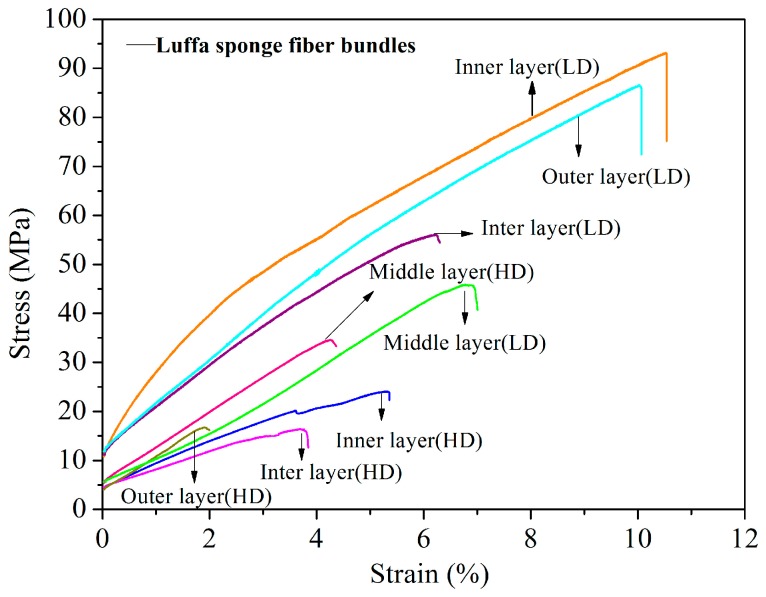
Typical stress–strain curves of fiber bundles in different layers taken from LD and HD luffa sponge.

The typical stress–strain curves for fiber bundles from *Arenga engleri*, jute, and four layers in luffa sponge were obtained (Figure 14). The curves correspond to data, that are in close proximity to the means. The curves show yielding, followed by plastic deformation, until breakage from 3% to 10% strain for fiber bundles of luffa sponge. Table 4 presents the mechanical properties of fiber bundles taken from *Arenga engleri*, jute, and four layers in luffa sponge. In comparison to fiber bundles in each layer from HD luffa sponge, the fiber bundles in corresponding layer from LD luffa sponge demonstrated higher tensile strength, Young’s modulus (excluding the middle layer), and elongation. In other words, the mechanical properties of single fiber bundles in LD luffa sponge were superior to HD luffa sponge. The phenomenon may be explained by the values of S_N_%, since the S_N_% of the fiber bundles in each layer (excluding middle layer) of HD luffa sponge were higher than the corresponding layer of LD luffa sponge. The presence of fibers predominantly contributes to the mechanical strength of the fiber bundles while the presence of cavities vessels and phloem tissue tends to reduce the mechanical strength [25]. In other words, higher the value of S_N_% in fiber bundles, lower is the amount of substantial fibers in fiber bundles, resulting in worse mechanical properties. In the case of same kind of luffa sponges, the mechanical properties of fiber bundles in each layer also decreased with the increase of S_N_% in fiber bundles. In addition, Table 5 shows Pearson’s correlations between the mechanical properties and the structural characteristics of luffa sponge fiber bundles. The results indicate that the Young’s modulus and the tensile strength have significant negative correlation with S_N_%. Therefore, the analysis of Pearson’s correlations further demonstrate that mechanical properties of luffa sponge fiber bundles decrease with the increase in S_N_%.

**Figure 15:**
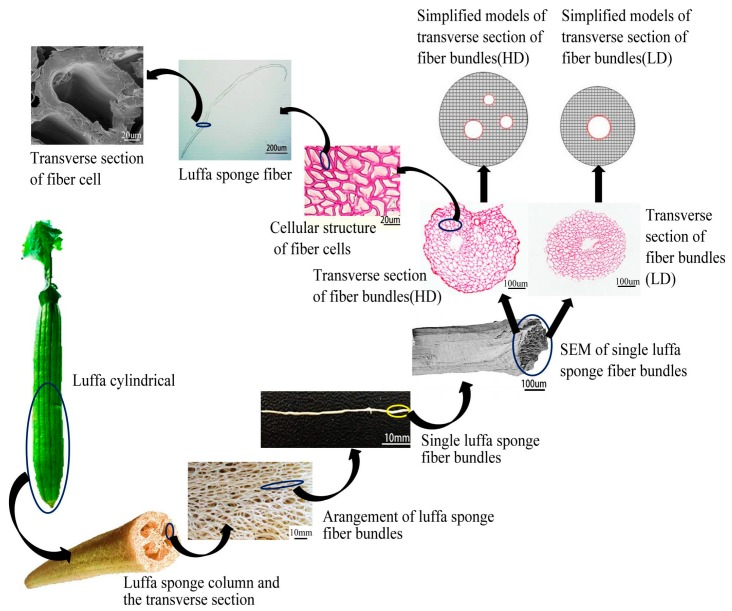
The hierarchical polyporous structure of luffa sponge (including luffa sponge column, luffa sponge fiber bundles, and luffa sponge fiber cells).

Moreover, the mechanical properties of fiber bundles from HD luffa sponge decreased dramatically with a slight increase in S_N_%. There are several factors responsible for these results, one of which is the presence and contribution of cavities in the transverse section of luffa sponge. The simplified models for transverse section of fiber bundles taken from two-density luffa sponge are shown in the Figure 15. As mentioned above, only one cavity was observed near the center in fiber bundles from LD luffa sponge, resulting in a circular distribution of fiber cells, while two or more cavities contributed at random in fiber bundles from HD luffa sponge, making the fiber cells unable to gather. The mechanical properties of the fiber bundles are a combination of the mechanical strength of all the fiber cells and the adhesion among fiber cells [37,38]. The aggregation degree of fiber has an important influence on the mechanical properties of fiber bundles. For instance, higher aggregation degree of fiber cells results in better mechanical properties of fiber bundles. This is one of the reasons for lower mechanical strength of fiber bundles from HD luffa sponge as compared to the ones from LD luffa sponge.

In addition, it is interesting to note that the density of fiber bundles taken from luffa sponge was in the range of 385.46–468.70 kg/m^3^ and was far lower than that of fiber bundles from *Arenga engleri* (950.20 kg/m^3^), and jute (1360.40 kg/m^3^). It is possible that the low density for luffa sponge fiber bundles is due to the hierarchical polyporous structure of fiber bundles and fiber cells as shown in Figure 15. Hence, luffa sponge is a low-density, light-weight natural fiber material. Although, the tensile strength of luffa sponge fiber bundles were lower than those of fiber bundles from *Arenga engleri* and jute, the specific modulus of luffa sponge fiber bundles were in a normal range. Specifically, the specific modulus of L.D. luffa sponge was higher than that of *Arenga engleri* fiber bundles. The natural plant fiber with higher mechanical strength and specific modulus, as the reinforcement of the composite material, can be used to manufacture the polymer matrix composite material with advantages of portability and good mechanical properties [39,40].

### 3.3. Scanning Electron Microscopy (SEM) Analysis

**Figure 16:**
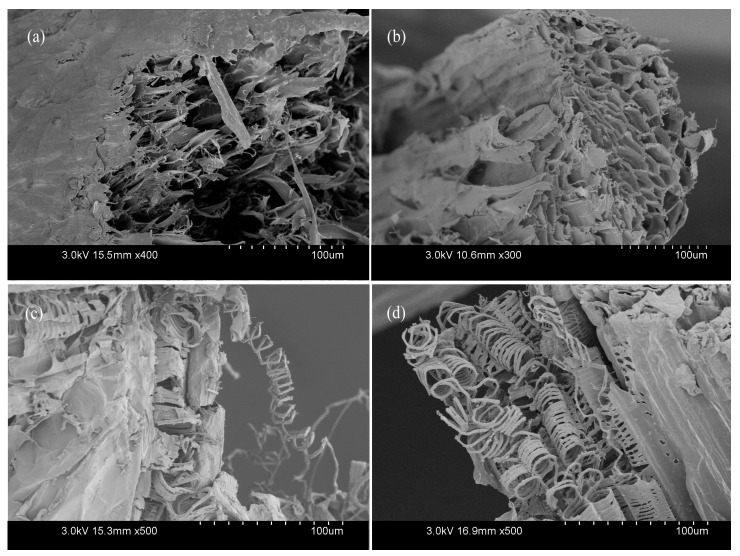
The fractured surface of fiber bundles taken from luffa sponges; (**a**,**c**) low density luffa sponge; and (**b**,**d**) high density luffa sponge.

To further understand the fracture mechanism, the fractured surface of broken fiber bundles was observed under scanning electron microscopy (SEM). As shown in Figure 16, fiber cell of LD luffa sponge (Figure 16a) had an obvious slip phenomenon at break, while the fractured surface of fiber cell of HD luffa sponge (Figure 16b) was flat. Since each fiber cell is connected with the surrounding material, when being stretched, external force has to overcome the strains of fiber cells and the adhesion stress among fiber cells. If the strength of fiber cells is superior to adhesion stress, the fiber cells survive, else the fiber cells break. In this regard, the strength of the fiber cells of luffa sponge is relatively high, and is the primary reason behind superior mechanical strength of fiber bundles from LD luffa sponge in comparison with HD luffa sponge.

Furthermore, as shown in Figure 16c,d, abundant spring-like spiral vessels were observed in the fiber bundles. It was observed that the behavior of pullout spiral vessels was similar to that of the spring lost elasticity.

**Figure 17:**
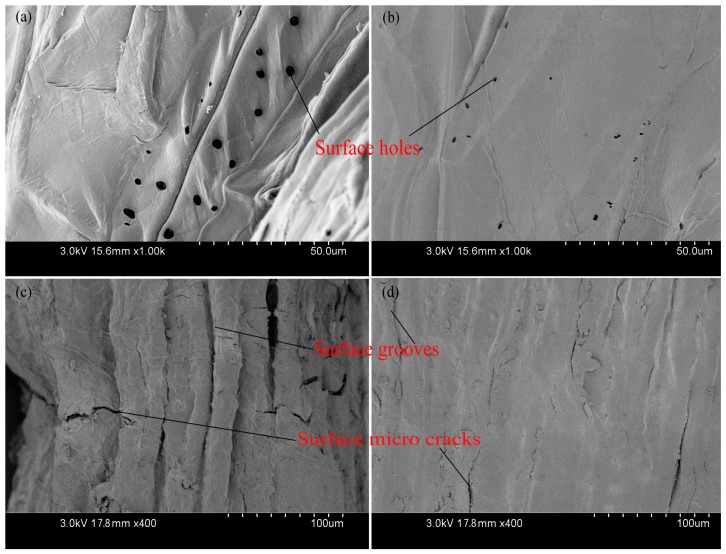
The surface topography of fiber bundles taken from luffa sponges: (**a**,**c**) low density luffa sponge; and (**b**,**d**) high density luffa sponge.

In Figure 17, representative surface topography of luffa sponge fiber bundles is presented. It was observed that fiber bundles from LD luffa sponge had more abundant grooves, holes, and micro cracks on the surface as compared to HD luffa sponge.

### 3.4. Moisture Regain

The moisture regain of fiber bundles from luffa sponge, *Arenga engleri*, and jute is presented in Table 6. The luffa sponge fibers exhibited a higher moisture regain as compared to *Arenga engleri* and jute. The higher moisture regain can possibly be attributed to the presence of the porous structure, surface groove, and micro cracks in luffa sponge fiber bundles. Tan et al. have discovered that slender hole exists in the bamboo fiber surface and indicated that these were important for fine moisture regain and moisture dispersion [41]. In comparison with the HD luffa sponge, LD luffa sponge fiber had higher moisture regain in the range of 10.2–10.9%, as compared to 7.1–9.3% for HD luffa sponge. There were slight differences in moisture regain among four layers in fiber from LD luffa sponge fiber, while those in the corresponding layers in H.D luffa sponge fiber were significant. In the case of HD luffa sponge fiber, the middle layer demonstrated highest moisture regain of 9.3%, while, the inner layer demonstrated the lowest moisture regain of 7.1%. Additionally, as shown in Figure 17, the grooves, holes, and micro cracks on the surface of the fiber bundles from LD luffa sponge were more abundant than those on HD luffa sponge. These results can be a possible explanation for higher moisture in LD luffa sponge fiber as compared to HD luffa sponge fiber. The moisture regain is an essential parameter as it affects the dimensional and physical properties of the fibers. The moisture regain influences the dimensional stability, electrical resistivity, tensile strength, porosity, and swelling behavior of natural fiber reinforced composites [42,43]. Accordingly, low moisture content of HD luffa sponge fiber is beneficial for fabricating luffa sponge fiber reinforced polymer composites due to their less ability to absorb water molecules.

### 3.5. X-ray Diffraction Analysis

Table 7 shows the relative crystallinity of luffa sponge, *Arenga engleri*, and jute. The relative crystallinity of each layer of LD luffa sponge was higher than that of corresponding layer in HD luffa sponge, leading to better mechanical strength of LD luffa sponge. Moreover, the trend of variation in relative crystallinity is in accordance with the variation trend of mechanical strength of luffa sponge, indicating that relative crystallinity had significant influence on the mechanical properties of luffa sponge. However, in comparison with *Arenga engleri* or jute, luffa sponge fiber bundles had higher relative crystallinity but lower mechanical properties. The possible reason could be the existence of cavities and smaller ratio of wall to lumen for luffa sponge than *Arenga engleri* or jute. The luffa sponge fibers, as natural fibers, have complex chemical components with a complex crosslinking of lignin and hemicellulose. All the above factors significantly affect the mechanical properties of luffa sponge fiber bundles.

### 3.6. TGA

**Figure 18:**
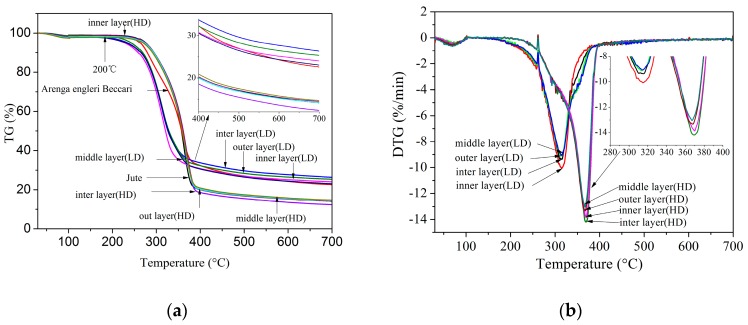
(**a**) Thermogravimetric analysis (TGA) curves of luffa sponge, *Arenga engleri* and jute; and (**b**) Thermogravimetric analysis and derivative thermogravimetric analysis (TGA–DTG) curves of different layer from luffa sponge of low density and high density.

Figure 18 and Table 8 show the TG and derivative thermogravimetric analysis (DTG) curves and data of luffa sponge, *Arenga engleri*, and jute. As shown in Figure 18, the TG curves of each layer of luffa sponge, *Arenga engleri* and jute were similar. The biomass combustion includes four main phases: water evaporation stage, the devolatilization and combustion stage, the fixed carbon combustion stage, and the burnout stage [44]. As shown in Figure 18, the degradation temperatures of each layer from LD luffa sponge were higher than those of corresponding layer from HD luffa sponge. In the case of HD luffa sponge, initial degradation temperature was in the range of 319.7–324.6 °C, final degradation temperature was in range of 384.8–387.4 °C, and the peak degradation temperature was in range of 366.3–369.9 °C, while those for LD luffa sponge were 269.6–271.3 °C, 309.8–350.8 °C, and 276.1–314.3 °C, respectively. It is generally agreed that higher the relative crystallinity, the better is the stability, including the thermal stability of fibers [45]. However, our results show that fiber from LD luffa sponge has higher relative crystallinity, but they demonstrate worse thermal stability as compared to HD luffa sponge. The difference in content of cellulose, hemicellulose, and lignin in both kinds of luffa sponge should be taken into consideration. Under normal circumstances, firstly, the hemicellulose degrades, followed by cellulose and lignin. Hence, the lignin content in HD luffa sponge could possibly be higher than LD luffa sponge. Furthermore, the thermal stability of HD luffa sponge is similar to jute and superior to *Arenga engleri*. In the case of jute, initial degradation temperature was 325.6 °C, final degradation temperature was 379.3 °C and the peak degradation was 365.7 °C, while those for *Arenga engleri* were 300.9 °C, 383.0 °C and 357.4 °C, respectively. In 2004, Tang et al. reported that, for the bamboo fibers, the initial degradation temperature is 342.6 °C, final degradation temperature is 380.4 °C and the peak degradation is 368.8 °C [41]. In 2016, Ridzuan et al. reported that the peak degradation of *Pennisetum purpureum* is 364.7 °C. Hence, in regards to thermal stability, it is evident that the fiber of HD luffa sponge is the preferred natural fiber material.

## 4. Conclusions

This study investigated the relationship between the structural characteristics and mechanical properties of luffa sponge fiber bundles, and their moisture regain and thermal performance as the reinforcement of lightweight composite. From the above discussions, the following conclusions can be drawn.

1. Luffa sponge fiber bundles are natural fiber materials of light texture with a density range of 385.46–468.70 kg/m^3^, which is significantly lower than that of *Arenga engleri* (950.20 kg/m^3^) and jute (1360.40 kg/m^3^). Furthermore, luffa sponge fiber bundles had excellent specific modulus. Specifically, specific modulus of luffa sponge fiber bundles of LD (2.07–4.05 MPa*m^3^/Kg) was even higher than that for *Arenga engleri*, depsite the fact that luffa sponge fiber bundles have lower tensile strength.
2. Special structural characteristics of luffa sponge fiber bundles had a significant impact on the mechanical properties. The phloem tissues of vascular bundle degenerated to cavities leading to a reduction in the mechanical strength of luffa sponge fiber bundles. Interestingly, only one cavity was observed in transverse section fiber bundles from LD luffa sponge, while two or more cavities were observed in fiber bundles from HD luffa sponge. The tensile tests proved that luffa sponge fiber bundles of HD possess lower mechanical strength than luffa sponge fiber bundles of LD. The mechanical properties of the fiber bundles were affected by the ratio of S_N_ and the number and contribution of cavities in the transverse section of luffa sponge fiber bundles. In other words, luffa sponge fiber bundles can have excellent mechanical properties if they have high ratio of S_N_, even-distribution, and high aggregation degree of fiber cell.
3. The smaller wall thickness of fiber cell and the cavity in the transverse section contributed to lower density of luffa sponge fiber bundles as compared to jute or *Arenga engleri* fiber bundles. The luffa sponge fiber cells had higher ratio of wall thickness to lumen than jute or *Arenga engleri*; in other words, the luffa sponge fiber cells had more substantive material. Accordingly, fiber bundles from HD luffa sponge should ideally possess great mechanical strength. However, this is in contrast with our results that fiber bundles from LD luffa sponge have superior tensile strength than that of HD luffa sponge. The probable reason for the discrepancy could be the high crystallinity of cellulose in LD luffa sponge.
4. The luffa sponge fiber bundles had relatively higher moisture regain in comparison with the common fibers. The possible explanation for the results could be the porous structure, the surface grooves, and the surface micro cracks in the luffa sponge fiber bundles. The moisture regain of HD luffa sponge fiber bundles (7.1–9.3%) was lower than that of LD luffa sponge fiber bundles (10.2–10.9%). Moreover, each layer fibers of luffa sponge had significantly different moisture regain in the case of HD luffa sponge while the corresponding difference was negligible in LD luffa sponge.
5. The biomass combustion of luffa sponge fiber consisted of four main phases: the water evaporation stage, the devolatilization and combustion stage, the fixed carbon combustion stage, and the burnout stage. There were remarkable differences in the thermal degradation temperatures between LD and HD luffa sponge fibers. The HD luffa sponge fiber demonstrated better thermal performance than LD luffa sponge fiber due to lower crystallinity of cellulose. These results are in contradiction with the widely accepted notion that the higher is the crystallinity of cellulose, the better is the thermal stability of fiber. The difference in the content of cellulose, hemicellulose, and lignin in two kinds of luffa sponge are considerable factors affecting thermal performance. The HD luffa sponge fiber has relatively more lignin.

## Figures and Tables

**Table 1:** Fiber cell characteristics of luffa sponge, *Arernge engleri* and jute.

		Fiber Cell of Luffa Sponge(LD)				Fiber Cell of Luffa Sponge(HD)				*Arenga engleri*	Jute
		Outer Layer	Inter Layer	Inner Layer	Middle Layer	Outer Layer	Inter Layer	Inner Layer	Middle Layer		
Length (um)	Mean	1359	1708	1610	1331	1594	1075	1488	1072	717	2331
	SD	430	450	482	398	503	349	602	458	14.98	36.14
Diameter (um)	Mean	25.25	22.29	20.78	24.35	22.37	17.32	26.53	22.65	14.14	19.6
	SD	6.57	6.48	5.19	7.23	6.34	3.5	7.08	5.71	0.33	0.13
Lumen diameter (um)	Mean	21.23	23.82	17.45	20.53	17.16	12.52	19.04	16.55	8.9	6.11
	SD	6.32	6.09	4.99	7.12	5.09	3.44	6.15	5.05	0.24	0.11
Wall thickness (um)	Mean	4.02	4.41	3.51	3.82	5.21	3.85	7.48	6.1	5.24	7.41
	SD	0.83	0.82	0.72	0.74	2.16	1.02	1.96	1.74	0.08	0.07
ratio of wall to lumen	Mean	0.19	0.18	0.2	0.18	0.3	0.29	0.39	0.36	0.59	1.21
	SD	0.06	0.05	0.07	0.07	0.12	0.10	0.15	0.17	0.01	0.05

Note that the SD is the Standard Deviation of data.

**Table 2:** Fiber bundles characteristic of luffa sponge (n = 20).

		Fiber Bundles of Luffa Sponge (LD)				Fiber Bundles of Luffa Sponge (HD)			
		Outer Layer	Inter Layer	Inner Layer	Middle Layer	Outer Layer	Inter Layer	Inner Layer	Middle Layer
S (100 µm^2^)	mean	1100	940	1277	1381	1819	1481	2000	1766
	SD	16	18	20	14	17	14	17	23
S_F_ (100 µm^2^)	mean	878	705	1109	1050	1307	1096	1568	1413
	SD	12	16	18	16	15	11	15	20
S_N_ (100 µm^2^)	mean	222	235	168	331	512	385	432	353
	SD	12	15	15	13	13	10	13	16
S_F_%	mean	80%	75%	87%	76%	72%	74%	78%	80%
	SD	2%	2%	3%	2%	3%	3%	2%	3%
S_N_%	mean	20%	25%	13%	24%	28%	26%	22%	20%
	SD	2%	2%	3%	2%	3%	2%	2%	3%

**Table 3:** Analysis of Variance (ANOVA) of fiber bundles characteristic index values of luffa sponge.

			Sum of Squares	df	Mean Square	F	Sig.
Fiber bundles of Luffa sponge (LD)	S	Between Groups	2,261,000	3	753800	2554.3	0.000
		Within Groups	22,430	76	295	-	-
		Total	2,284,000	79	-	-	-
	S_N_%	Between Groups	0.173	3	0.058	391.674	0.000
		Within Groups	0.011	76	0.000	-	-
		Total	0.184	79	-	-	-
Fiber bundles of Luffa sponge (HD)	S	Between Groups	2,773,758	3	924586	117.359	0.000
		Within Groups	598,750	76	7878	-	-
		Total	3,372,507	79	-	-	-
	S_N_%	Between Groups	0.853	3	0.028	284.974	0.000
		Within Groups	0.008	76	0.000	-	-
		Total	0.093	79	-	-	-
Fiber bundles of Luffa sponge (LD-HD)	S	Between Groups	14,035,893	1	14035893	392.073	0.000
		Within Groups	5,656,266	158	35799	-	-
		Total	19,692,159	159	-	-	-
	S_N_%	Between Groups	0.047	1	0.047	26.655	0.000
		Within Groups	0.277	158	0.002	-	-
		Total	0.324	159	-	-	-

**Table 4:** Mechanical properties of single fiber bundles in different layers taken from two-density luffa sponge and single fiber bundles of *Arernga engleri* and jute (n = 50).

		Density (kg/m^3^)		Young’s Modulus (MPa)		Specific Modulus (MPa·m^3^/Kg)		Tensile Strength ^a^ (MPa)		Elongation (%)	
		Mean	SD	Mean	SD	Mean	SD	Mean	SD	Mean	SD
Luffa sponge (LD)	outer layer	458.31	2.37	1208	145.9	2.64	1.54	73.46	34.25	4.98	3.94
	inter layer	431.85	1.68	958	236.5	2.22	1.23	53.82	17.42	5.16	3.95
	inner layer	468.70	0.60	1897	320.1	4.05	1.24	91.63	14.32	9.81	3.19
	middle layer	443.80	3.69	918	245.9	2.07	0.78	61.80	24.22	6.27	2.43
Luffa sponge (HD)	outer layer	391.50	0.93	635	82.09	1.62	0.56	24.03	3.12	2.54	0.39
	inter layer	385.46	1.22	555	193.1	1.44	0.43	24.31	8.8	3.2	1.62
	inner layer	411.73	1.91	1133	299.8	2.75	1.03	39.91	11.94	3.73	1.19
	middle layer	421.90	1.69	880	577.2	2.09	0.85	42.55	8.18	6.67	3.70
*Arenga engleri*		950.20	0.90	1735	187.5	1.83	0.58	102.34	13.18	15.76	3.58
Jute		1360.40	0.40	25119	530.9	18.46	3.21	378.59	113.14	0.83	0.25

^a^ Excluding the S_N_ area and recalculating tensile strength using the effective area S_F_.

**Table 5:** Pearson’s correlation between mechanical properties and characteristic of luffa sponge.

		Young’s Modulus	Tensile Strength	Elongation
S	Pearson Correlation	0.270	0.284	0.344 *
	Sig(2-tailed)	0.092	0.076	0.030
	N	40	40	40
S_N_	Pearson Correlation	−0.373 *	−0.201	−0.008
	Sig(2-tailed)	0.018	0.214	0.959
	N	40	40	40
S_F_	Pearson Correlation	0.420 **	0.350 *	0.351 *
	Sig(2-tailed)	0.007	0.0270	0.026
	N	40	40	40
S_N_%	Pearson Correlation	−0.552 **	−0.409 **	−0.253
	Sig(2-tailed)	0.000	0.009	0.116
	N	40	40	40
S_F_%	Pearson Correlation	0.552 **	0.409 **	0.253
	Sig(2-tailed)	0.000	0.009	0.116
	N	40	40	40

* Correlation is considered significant at 0.05 level. ** Correlation is considered significant at 0.01 level.

**Table 6:** The moisture regain of luffa sponge fibers (n = 60).

		Luffa Sponge (LD)				Luffa Sponge (HD)				*Arenga engleri*	Jute
		Outer Layer	Inter Layer	Inner Layer	Middle Layer	Outer Layer	Inter Layer	Inner Layer	Middle Layer		
Moisture regain (%)	mean	10.4	10.2	10.2	10.9	8.9	7.1	9.3	8.8	8.0	7.3
	SD	0.2	0.4	0.2	0.3	0.4	0.4	0.4	0.3	0.4	0.4

**Table 7:** The relative crystallinity of luffa sponge, *Arenga engleri*, and jute (n = 3).

		Luffa Sponge (LD)				Luffa Sponge (HD)				*Arenga engleri*	Jute
		Outer Layer	Inter Layer	Inner Layer	Middle Layer	Outer Layer	Inter Layer	Inner Layer	Middle Layer		
Relative crystallinity (%)	mean	42.3	37.7	47.1	30.1	39.0	33.1	39.6	24.4	29.0	31.8
	SD	0.9	0.8	1.1	0.5	0.6	0.3	1.1	0.7	0.8	1.0

**Table 8:** Thermogravimetric analysis of fibers taken from luffa sponge, *Arenga engleri*, and jute (n = 3).

		Initial Degradation Temperature/°C		Final Degradation Temperature/°C		Peak Degradation/°C		Final Residue/%	
		Mean	SD	Mean	SD	Mean	SD	Mean	SD
Luffa sponge (LD)	outer layer	269.6	3.4	309.8	4.2	276.1	2.6	32.7	0.4
	inter layer	270.2	2.2	346.1	3.5	314.1	3.1	35.1	0.6
	inner layer	270.3	2.5	344.6	4.3	307.8	3.8	31.9	1.2
	middle layer	271.3	2.8	350.8	2.6	314.3	2.3	34.0	0.8
Luffa sponge (HD)	outer layer	321.4	2.3	384.8	3.7	368.3	4.3	19.2	0.5
	inter layer	321.9	2.6	387.4	4.1	366.3	4.1	18.7	1.0
	inner layer	324.6	3.0	386.5	3.4	369.9	4.3	19.2	0.7
	middle layer	319.7	3.2	385.7	2.7	367.0	3.7	21.0	1.0
*Arenga engleri*		300.9	4.1	383.0	3.4	357.4	4.6	31.0	0.8
Jute		325.6	4.3	379.3	3.9	365.7	3.1	19.6	0.6
